# Adaptive satellite attitude control for varying masses using deep reinforcement learning

**DOI:** 10.3389/frobt.2024.1402846

**Published:** 2024-07-23

**Authors:** Wiebke Retagne, Jonas Dauer, Günther Waxenegger-Wilfing

**Affiliations:** ^1^ Institute of Space Propulsion, German Aerospace Center (DLR), Hardthausen, Germany; ^2^ Department of Physics, Technische Universität Darmstadt, Darmstadt, Germany; ^3^ Institute of Computer Science, University of Wuerzburg, Wuerzburg, Germany

**Keywords:** attitude control, deep reinforcement learning, adaptive control, spacecraft dynamics, varying masses, space debris, active debris removal

## Abstract

Traditional spacecraft attitude control often relies heavily on the dimension and mass information of the spacecraft. In active debris removal scenarios, these characteristics cannot be known beforehand because the debris can take any shape or mass. Additionally, it is not possible to measure the mass of the combined system of satellite and debris object in orbit. Therefore, it is crucial to develop an adaptive satellite attitude control that can extract mass information about the satellite system from other measurements. The authors propose using deep reinforcement learning (DRL) algorithms, employing stacked observations to handle widely varying masses. The satellite is simulated in Basilisk software, and the control performance is assessed using Monte Carlo simulations. The results demonstrate the benefits of DRL with stacked observations compared to a classical proportional–integral–derivative (PID) controller for the spacecraft attitude control. The algorithm is able to adapt, especially in scenarios with changing physical properties.

## 1 Introduction

With increased access to space, the challenge of space debris is becoming an increasingly relevant topic. To keep space useable for future generations, space debris must be mitigated. The most effective mitigation measure includes active debris removal (ADR) of approximately five objects per year. This research investigates the challenge of the attitude control system (ACS) in ADR. The difficulty here is that often, the exact size, shape, and mass of the targeted debris objects are unknown. Furthermore, through the capturing process, the mass and inertia of the satellite system are changed, making it difficult for the ACS to maintain a stable position. In spacecraft attitude control, proportional–integral–derivative (PID) control remains a cornerstone due to its simplicity and computational efficiency (Show et al., 2002). For more complex, nonlinear attitude problems, more sophisticated algorithms, such as model predictive control or dynamic programming, can be used. Model predictive control implements a predictive model of the system to anticipate future behavior over a finite time horizon (Iannelli et al., 2022). The control inputs are optimized by minimizing a specific cost function. This generally performs better than PID algorithms but also requires more computational resources. In this research, a deep reinforcement learning (DRL) approach is investigated to solve this satellite attitude problem. DRL has the benefit of being very computationally efficient once the networks are trained. During the process of an ADR, the ACS system must handle the unknown and changing mass. To simulate this, the agent is trained with varying masses and must develop an appropriate control strategy using torques on the reaction wheels of the satellite. This application of DRL to the satellite attitude problem is not new and has shown great promise in the past. An overview can be found in Tipaldi et al. (2022). The partially known space environment, the time-varying dynamics, and the benefits of autonomy make it the perfect candidate for reinforcement learning (RL). Research on this topic has been mainly constricted to training the agent to develop a general algorithm independent of the inertia of the satellite. Allison et al. (2019) investigated the use of a proximal policy optimization (PPO) algorithm to find an optimal control strategy for the spacecraft attitude problem. The spacecraft was modeled as a rigid body, and the state space was defined by the error quaternion vectors. Even though the controller was trained with a single mass, the control policy obtained was able to achieve good performance for a range between 0.1 kg and 100,000 kg. Specifically, the DRL approach is suitable to develop a general solution that can handle a wide array of different masses. Although a general solution is suitable for many applications, some maneuvers require adaptability in attitude control. In an ADR scenario, the mass of the spacecraft system changes drastically, both after capture and after the release of the debris object. Due to the nature of the general approach, it is limited by the edge cases of very small or very high masses. An algorithm developed to perform optimally for masses between 500 kg and 1,000 kg might develop problems at very low masses, applying forces that are too large. In the ADR scenario, considerable research effort has been devoted to the rendezvous maneuver and the pre-docking proximity control. Xu et al. (2023) investigated a DRL approach to optimize rendezvous trajectories for fuel consumption. It is shown that the DRL approach is suitable for a large number of impulses. Jiang et al. (2023) solved an interception strategy with high uncertainty using a one-to-one DRL algorithm. The algorithm can develop a universal interception strategy, even in random environments. These works demonstrate the suitability of DRL for changing conditions and uncertain conditions. This work instead focuses on the scenario after the capture, where a detumbling must be performed. In this scenario, the mass of the spacecraft system changes drastically, both after capture and after the release of the debris object. This research aims to develop an approach that adapts its control strategy depending on the mass of the satellite. Because the mass of the spacecraft system cannot be measured directly, the algorithm must extract this from other measurements. The authors compare different strategies and investigate whether they improve the performance of the algorithm, especially for the previously described edge cases. In particular, stacked observations are employed. Stacked observations are the result of stacking several observations into one single state. The authors show that all control strategies effectively detumble the system. Controllers that cannot extract the dynamics of a system struggle to handle situations in which the masses are very low. In contrast, RL agents with stacked observations can master the situation even with small masses. A more detailed description of the theory behind the developed algorithms can be found in Section 2. The methodology needed to reproduce the results is presented in Section 3. In addition, the robustness of each approach is examined using a Monte Carlo analysis. The agents are presented with different scenarios. The analyzed strategies are then compared to a classical PID controller, a well-established feedback control strategy, in Section 4.

## 2 Theory

This chapter provides the necessary theoretical background for the spacecraft attitude control problem. Furthermore, an introduction to deep reinforcement learning and the applied algorithms is given, and the spacecraft attitude problem is described in detail.

### 2.1 Dynamic framework

#### 2.1.1 Coordinate frames

Before specifying the spacecraft dynamics, a common reference frame needs to be defined. Two different coordinate frames are used in the simulation. The first one is the **earth-centered inertial frame (ECI)** N . The center of its origin is at the center of the earth. For inertial coordinate frames, a fixed reference direction must be defined. Here, the vernal equinox is used. This describes the line of intersection between the equator and the ecliptic plane. The sun passes this point twice per year. Following the specification given by Vallado and McClain (2001) in the ECI, the *I* axis points toward the vernal equinox, the *J* axis is 90°C to the east in the equatorial plane, and the *K* axis extends through the North pole. Another coordinate frame used is the **spacecraft body frame** B . According to Alcorn et al. (2018), this describes a reference frame that is fixed to the rigid body of the spacecraft. The origin of the frame is denoted as *B*. *B*_*c*_ describes the center of mass of the hub. $r_{B/N}$ describes the position of the spacecraft in reference to the inertial ECI frame N . The angular velocity $\omega_{B/N}$ between the ECI N and the spacecraft body frame B is given by Eq. 1.

$$\omega_{B/N}={\left[\omega_{1},\omega_{2},\omega_{3}\right]}^{T}.$$ (1)

#### 2.1.2 Spacecraft dynamics

The spacecraft is modeled as a rigid body. The satellite is equipped with three balanced reaction wheels, each aligned with one spin axis ${\hat{g}}_{s_{i}}$ . This enables each reaction wheel to control one spin axis without influencing the others. The equation of motion (EoM) used to model the rotational behavior in the simulation is displayed in Eqs 2, 3:

$$m_{sc}\left[\tilde{c}\right]{\ddot{r}}_{B/N}+\left(\left[I_{sc,B}\right]-\overset{3}{\underset{i=1}{\sum }}J_{s_{i}}{\hat{g}}_{s_{i}}{\hat{g}}_{s_{i}}^{T}\right){\dot{\omega }}_{B/N},$$ (2)

$$\begin{array}{l} \;=-\left[{\tilde{\omega }}_{B/N}\right]\left[I_{sc,B}\right]\omega_{B/N}\\ \;-\overset{3}{\underset{i=1}{\sum }}\left({\hat{g}}_{s_{i}}u_{s_{i}}+\omega_{B/N}\times J_{s_{i}}\Omega_{i}{\hat{g}}_{s_{i}}\right)-\left[I_{sc,B}^{\prime }\right]\omega_{B/N}. \end{array}$$ (3)

The tilde operator designates the skew symmetric matrix, that is, $\left[\tilde{\omega }\right]v=\omega \times v$ . This notation has the benefit of being frame-independent and represents the cross product in a compact manner. A more detailed description can be found in Alcorn et al. (2018) and Schaub and Junkins (2018). The contributions to the EoM are summarized in Table 1.

**TABLE 1:** Contributions to the EoM for the spacecraft equipped with reaction wheels. The EoM depends heavily on the mass and inertia of the spacecraft. The attitude rate and the wheel speed influence the effect the motor torque has on the spacecraft.

Contributions to the equation of motion	
Inertial forces on the center of mass *c* of the spacecraft (SC).	$\left[m_{sc}\tilde{c}\right]{\ddot{r}}_{B/N}$
Inertia matrix of the SC and the reaction wheels (RWs)	$\left[I_{sc,B}\right],J_{s_{i}}$
Attitude rate of the SC	$\omega_{B/N}$
Combined torques generated by the RWs	
Effect of the motor torque	$u_{s_{i}}$
Effect of the attitude rate	$\omega_{B/N}$
Effect of the wheel speed	**Ω**

### 2.2 Deep reinforcement learning

The reinforcement learning problem can be characterized through the interaction of the agent with an environment. The agent is the learner and decision maker, while the environment provides the background and gives feedback to those decisions. The agent interacts with the environment in a discrete time step *t* through an action *a*_*t*_. Afterward, the agent receives feedback in the form of the subsequent state *s*_*t*+1_, which contains information about the environment. To evaluate the action, the agent also receives a numerical reward *r*_*t*+1_. The goal is to maximize the sum of discounted rewards in the long run. The sum of these rewards *r* is called the return *R*. Mathematically, the RL problem can be represented as a Markov decision process (MDP). If an environment satisfies the Markov property, it generally means the present state is only dependent on the immediate previous state. The MDP is defined by Eqs 4–9

$$MDP≔\left(S,A,T,P,r\right)$$ (4)

with

S≔State space, (5)

A≔Action space, (6)

T⊆N≔Set of time steps, (7)

$$P:S\times A\times T\times S\to \left[0,1\right]≔\text{State}-\text{transition probability function},$$ (8)

r:S×A×T×S→R≔Reward function. (9)

The core RL objective is the maximization of the expected discounted return. A discount factor *γ* is used to weigh the future rewards and, therefore, determine how these are considered. The return R_t_ can then be written according to Eq. 10.

$$R_{t}=r_{t+1}+\gamma r_{t+2}+\gamma^{2}r_{t+3}+\cdots =\overset{\infty }{\underset{k=0}{\sum }}\gamma^{k}r_{t+k+1},\text{with }0\leq \gamma <1.$$ (10)

A policy *π*(*a*_*t*_, *s*_*t*_) is defined as the probability of selecting action *a*_*t*_ in state *s*_*t*_. To evaluate the policy, the state-value function Eq. 11 is defined. Informally, this is the expected discounted return when starting in state *s*_*t*_ and following a policy *π*. From this, the action-value function Eq. 12 is defined. This is the expected return when starting in state *s*_*t*_ and taking action *a*_*t*_ and from there on following policy *π*

$$\begin{array}{ll} V^{\pi }\left(s_{t},a_{t}\right)=E_{\pi }\left[R_{t}|S=s_{t}\right] & =E_{\pi }\left[\overset{\infty }{\underset{k=0}{\sum }}\gamma^{k}r_{t+k+1}|S=s_{t}\right], \end{array}$$ (11)

$$\begin{array}{ll} Q^{\pi }\left(s_{t},a_{t}\right) & =E_{\pi }\left[R_{t}|S=s_{t},A=a_{t}\right]\\ & =E_{\pi }\left[\overset{\infty }{\underset{k=0}{\sum }}\gamma^{k}r_{t+k+1}|S=s_{t},A=a_{t}\right]. \end{array}$$ (12)

The state and the action-value function can be defined recursively. These recursive definitions are called the Bellmann and are displayed in Eqs 13 and 14:

$$V^{\pi }\left(s_{t},a_{t}\right)=E_{a_{t}∼\pi \left(\cdot |s_{t}\right),s_{t+1}∼P\left(\cdot |s_{t},a_{t}\right)}\left(r_{t+1}+\gamma V^{\pi }\left(s_{t+1}\right)\right),$$ (13)

$$\begin{array}{ll} Q^{\pi }\left(s_{t},a_{t}\right) & =E_{s_{t+1}∼P\left(\cdot |s_{t},a_{t}\right)}\\ & \;\times \left(r_{t+1}+\gamma E_{a_{t+1}∼\pi \left(\cdot |s_{t+1}\right)}\left(Q^{\pi }\left(s_{t+1},a_{t+1}\right)\right)\right). \end{array}$$ (14)

The expected return *J*(*π*) associated with a policy is displayed in Eq. 15

$$J\left(\pi \right)=E_{t∼P\left(\cdot |\pi \right)}\left\{R_{t}\right\}.$$ (15)

The reinforcement learning problem can then be written in Eq. 16 as

$$\pi^{*}=\arg\underset{\pi }{\max}J\left(\pi \right),$$ (16)

where *π* ^*^ describes the optimal policy.

### 2.3 Soft actor-critic algorithm

The soft actor-critic (SAC) (Haarnoja et al., 2018) algorithm is an off-policy maximum entropy deep reinforcement learning algorithm. The goal is to maximize the expected reward and the entropy of the selected actions. SAC makes use of three major concepts.

#### 2.3.1 Actor-critic algorithm

Actor-critic algorithms are usually based on policy iteration. Policy iteration describes the alternation between policy evaluation and policy improvement. In the policy evaluation step, the Q-value function (critic) is updated on the basis of the actor. In the policy improvement step, the parameters Φ of the policy *π*_Φ_ are updated with the critic.

#### 2.3.2 Off-policy algorithm

The off-policy paradigm helps increase sample efficiency through the reuse of previously collected data. Off-policy algorithms have the advantage of distinguishing between the current policy and the behavioral policy. The current policy can be updated with collected transitions (*s*_*t*_, *a*_*t*_, *r*_*t*_, *s*_*t*+1_) that are sampled by another policy. Therefore, it is possible to use data that were collected by an old policy to update the current policy. The SAC algorithm makes use of an experience replay buffer, which is a set D of previous experience.

#### 2.3.3 Entropy maximization

Entropy is a metric that describes the randomness of a random variable (Open AI, 2018b). The entropy H of a random variable *x* with probability or density function *P* is defined in Eq. 17.

$$H\left(P\right)=E_{x∼P}\left[-\log P\left(x\right)\right].$$ (17)

Entropy-regularized reinforcement learning extends the objective function by an entropy objective $H\left(\pi \left(\cdot |s_{t}\right)\right)$ . The expected return is then rewritten according to Eq. (18).

$$J\left(\pi \right)=\overset{T}{\underset{k=0}{\sum }}E_{\left(s_{t},a_{t}\right)∼\rho_{\pi }}\left[r\left(s_{t},a_{t}\right)+\alpha H\left(\pi \left(\cdot |s_{t}\right)\right)\right],$$ (18)

where *α* describes the temperature parameter. This determines the relative importance of the entropy term against the reward term. Therefore, it controls the stochasticity of the optimal policy. The extension by the entropy objective has the advantage of incentivizing the policy to explore the state-action space and make the policy more robust.In practice, SAC uses neural networks as function approximators for the parameterized soft Q-function *Q*_*θ*_(*s*_*t*_, *a*_*t*_) and the tractable policy *π*_Φ_(*a*_*t*_, *s*_*t*_), where *θ* and Φ describe the parameters of the neural networks. The soft Q-function is trained by minimizing the soft Bellmann residual error, displayed in Eq. 19.

$$\begin{array}{ll} J_{Q}\left(\theta \right) & =E_{\left(s_{t},a_{t}\right)∼D}\left[\frac{1}{2}\left(Q_{\theta }\left(s_{t},a_{t}\right)-\left(r\left(s_{t},a_{t}\right)\right.\right.\right.\\ & \;+\left.{\left.\left.\gamma E_{s_{t+1}∼p}\left[V\left(s_{t+1}\right)\right]\right)\right)}^{2}\right]. \end{array}$$ (19)

The value function Eq. 20 is implicitly parameterized through the soft Q-function

$$V\left(s_{t}\right)=E_{a_{t}∼\pi }\left[Q_{\bar{\theta }}\left(s_{t},a_{t}\right)-\alpha \log\pi \left(a_{t},s_{t}\right)\right].$$ (20)

The target network function, denoted by $Q_{\bar{\theta }}$ , is introduced to stabilize training in the algorithm. In order to achieve this, a second network is used, which is updated through the use of polyak averaging, a technique that involves computing a moving average of the Q-network. This is updated once per update of the main network and lags behind it. To prevent overestimation of the Q-values, two Q-functions are trained independently, and the minimum of their respective target networks is used to update the actor.The parameters *ϕ* of the policy network are updated by minimizing the return in Eq. 21.

$$J_{\pi }\left(\phi \right)=E_{s_{t}∼D}\left[E_{a_{t}∼\pi_{\psi }}\left[\alpha \log\left(\pi_{\psi }\left(a_{t}|s_{t}\right)\right)-Q_{\theta }\left(a_{t},s_{t}\right)\right]\right].$$ (21)

### 2.4 Proximal policy optimization

Proximal policy optimization (PPO) (Schulman et al., 2017; Open AI, 2018a) is an often-used reinforcement learning algorithm. The core idea of PPO is to take policy updates that are as far apart as possible without causing the performance to diverge. PPO is an actor-critic on-policy algorithm.

#### 2.4.1 On-policy algorithm

An on-policy algorithm iterates through two phases. At first, the agent samples data. In the second step, the agent updates its policy with the sampled data. Because the samples need to be generated with the policy that should be updated, all the training data are obsolete after an update has been performed. The agent must sample data again after every update.PPO updates its policy with an advantage function *A*(*s*_*t*_, *a*_*t*_); such as the ones given in Eqs 22 and 23.

$$A\left(s_{t},a_{t}\right)=Q\left(a_{t},s_{t}\right)-V\left(s_{t}\right)$$ (22)

$$=\left(r\left(s_{t},a_{t}\right)+\gamma V\left(s_{t+1}\right)\right)-V\left(s_{t}\right).$$ (23)

The advantage function *A*(*s*_*t*_, *a*_*t*_) provides a measure of how good action *a*_*t*_ is compared to the expected value of being in state *s*_*t*_. Because *A*(*s*_*t*_, *a*_*t*_) can be defined by the value function *V*(*s*_*t*_), PPO uses an actor network and a value function network. The value network is trained using the Bellmann equation. The policy is updated via Eq. 24.

$$\theta_{k+1}=\arg\underset{\theta }{\max}E_{\left(s_{t},a_{t}\right)∼\rho_{\pi }}\left[L\left(s_{t},a_{t},\theta_{k},\theta \right)\right].$$ (24)

Here, *L* is given in Eqs 25 and 26.

$$\begin{array}{ll} & L\left(s_{t},a_{t},\theta_{k},\theta \right)\\ & \;=\min\left(\frac{\pi_{\theta }\left(a_{t}|s_{t}\right)}{\pi_{\theta_{k}}\left(a_{t}|s_{t}\right)}A^{\pi_{\theta_{k}}}\left(s_{t},a_{t}\right),\;g\left(\epsilon ,A^{\pi_{\theta_{k}}}\left(s_{t},a_{t}\right)\right)\right), \end{array}$$ (25)

where

$$g\left(\epsilon ,A\right)=\left\{\begin{array}{cc} \left(1+\epsilon \right)A,\; & A\geq 0\\ \left(1-\epsilon \right)A,\; & A<0 \end{array}\right..$$ (26)

The hyperparameter *ϵ* controls the deviation between the old and the new policy and is, therefore, responsible for ensuring the update steps are not too large.

### 2.5 Stacked observations

Reinforcement learning is often influenced by hidden variables. Hidden variables are properties of the environment that are not present in the state of the RL problem but that have an influence on the dynamics of the system, such as the mass of an object. Although the hidden variables are not part of the state, the agent must be able to derive them in order to control the system optimally. In this work, we use stack past observations and actions to make the system dynamics accessible to the agent. Using this metric, the agent can analyze the historical states to infer details about how the system’s behavior changes over time.

## 3 Methodology

This section describes the simulation setup and the application of the RL algorithms to the spacecraft attitude problem.

### 3.1 Simulation architecture

The attitude problem is simulated within the astrodynamic framework Basilisk (2024). This framework provides Python modules written in C/C++. It is being developed by the University of Colorado Autonomous Vehicle Systems (AVS, 2023) and the Laboratory for Atmospheric and Space Physics (LASP, 2023). The simulation environment has no aerodynamic, gravitational, or solar radiation pressure effects. The simulations are visualized by the accompanying software, Vizard (2023). The simulation software is connected to the RL framework RLlib (2023) through OpenAI Gym.

**FIGURE 1:**
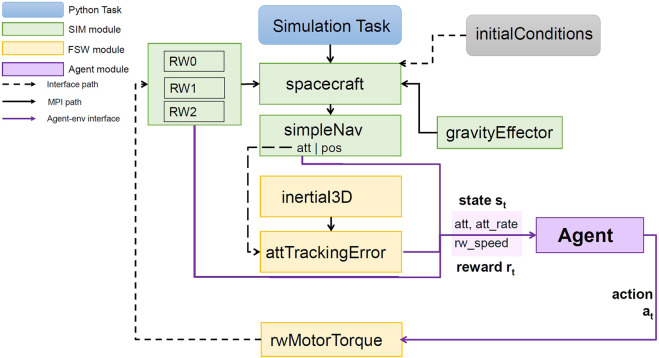
Simulation architecture and agent interface. The simulation (SIM) and flight software (FSW) modules communicate through the message-passing interface (MPI). The agent receives the state *s*_*t*_ from the environment. The state contains the attitude $\sigma_{B/N}$ , the attitude rate $\omega_{B/N}$ , and the wheel speed **Ω**. The agent then gives an action *a*_*t*_ in the form of a torque vector into the environment. The communication between the agent and the environment is based on an OpenAI Gym interface. Modified from Schaub (2023).

The simulation architecture is shown in Figure 1. The *spacecraft* module describes the spacecraft as a rigid body. The *gravityEffector* module provides the necessary orbital dynamics for a low earth orbit. The *simpleNav* module provides the current attitude, attitude rate, and position of the spacecraft. The *inertial3D* module provides the attitude goal. In the *attTrackingError* module, this goal is used to compute the difference between the desired attitude and the current attitude. The result is then given as feedback to the agent. The agent provides a torque input to the *rwMotorTorque* module, which maps these onto the three reaction wheels. The reaction wheel speed Ω is then given as feedback to the agent. The different scenarios described in the following sections were implemented through the *initialConditions* module. The agent and the simulation communicate through an OpenAI Gym interface. The agent is trained using the SAC (2023) and PPO (2023) algorithms included in RLlib.

### 3.2 State and action space

A satellite equipped with reaction wheels used for attitude control is simulated. Three reaction wheels are set up to control the three axes of the satellite. The attitude tracking error $\sigma_{B/N}$ and the attitude rate $\omega_{B/N}$ are described by modified Rodrigues parameters (MRP) Schaub and Junkins (2018). The resulting attitude is always given as the shortest rotational path. The MRP have a bounded maximum norm of 1, which allows the interpretation of the feedback gained from the attitude error. As action **a**, the torque vector *u* on the reaction wheels is used. The final action vector is then given in Eq. 27.

$$a=\left[u_{1},u_{2},u_{3}\right].$$ (27)

The reaction wheels are modeled after a standard set of reaction wheels like the Honeywell (2023) HR16. In compliance with this, the action space boundaries are set to *u*_min_ = −0.2 Nm and *u*_max_ = 0.2 Nm. The state **s** is defined with the reaction wheel speed **Ω** in the following Eq. 28.

$$s=\left[\sigma_{B/N},\omega_{B/N},\Omega \right].$$ (28)

### 3.3 Training

In the training scenario, a fixed initial attitude $\sigma_{B/N\text{Init}}=\left[1,0,0\right]$ (rotation by 180° around the *x*-axis) and attitude rate $\omega_{B/N\text{Init}}=\left[0,0,0\right]\frac{\text{rad}}{\text{s}}$ are set. The goal state is $\sigma_{B/N\text{Desired}}=\left[0,0,0\right]$ . The mass is sampled uniformly between 10 kg and 1,000 kg.

### 3.4 Reward modeling and RL parameters

The main objective of the agent is to reach the desired orientation. For this purpose, an attitude error is defined on which the reward is based. The attitude error $\sigma_{B/N}$ is defined in Eqs 29 and 30 as the difference between the desired orientation and the current orientation of the satellite for each coordinate.

$$\sigma_{B/N}=\left[\sigma_{B/N}^{1},\sigma_{B/N}^{2},\sigma_{B/N}^{3}\right],\text{ with }$$ (29)

$$\sigma_{B/N}^{i}=\left(\sigma_{B/N\text{Desired}}^{i}-\sigma_{B/N\text{Current}}^{i}\right).$$ (30)

Using this definition, the reward is defined in Eq. 31.

$$r=\left\{\begin{array}{c} -1,\text{ if }|\Omega_{i}|\geq \Omega_{\max}=6000\;\text{rpm}\;\\ -|\sigma_{B/N}{|}^{2}+0.1,\text{ if }\sigma_{B/N}^{i}<0.05,\;\forall i\;\\ -|\sigma_{B/N}{|}^{2}+0.05,\text{ if }\sigma_{B/N}^{i}\text{ and }\sigma_{B/N}^{j}<0.05,\;\forall i\neq j\;\\ -30\text{ and episode over},\text{ if }\omega_{B/N}^{i}>0.85\;\frac{\text{rad}}{\text{s}},\;\forall i\;\\ -|\sigma_{B/N}{|}^{2},\text{ else.}\; \end{array}\right.$$ (31)

The first term of the reward function is to avoid saturation of the reaction wheels at their maximum wheel speed Ω_max_. The main objective is to minimize the norm of the attitude error $-|\sigma_{B/N}{|}^{2}$ . Empirically, it could be observed that these two conditions alone do not achieve the desired orientation in each coordinate because the norm of the attitude error is in a similar small range, even if only one coordinate is near the desired orientation. Therefore, conditions were added, which consider the coordinates separately and give additional incentive to the agent to bring all coordinates below a certain threshold. These are represented in the second and third rows. The condition in the second row gives an additional reward if all coordinates are below 0.05. The condition in the third row gives a smaller additional reward if at least two coordinates are below the threshold. The fourth row prevents the satellite from spinning out of control. Especially with small masses, exerting a large torque can cause the satellite to spin uncontrollably, making further attitude control impossible. With −1 ≤ *r* ≤ 0.1 and the number of steps = 60, the minimum and maximum return *R* can be calculated to −60 ≤ *R* ≤ 6. The reward function is the same throughout all scenarios. The RL hyperparameters used for the training are shown in Tables 2 and 3. The training of the SAC agent was carried out on a desktop computer with a GeForce RTX 3090 GPU and an Intel Core i9-12900 KF CPU. A GeForce RTX 3090 GPU with an Intel Xeon Gold 6140 CPU was used for the PPO agent.

**TABLE 2:** Training parameters for the SAC algorithm, including the RL hyperparameters and the environment parameters. The learning rates refer to three different learning rates: The actor network, the Q-value network, and the *α* parameter. The rectified linear unit (ReLU) is chosen as the activation function. This function returns the input if the input is positive and zero otherwise.

Parameter	Value
Learning rates	3 ⋅ 10^–4^
Discount *γ*	0.99
Replay buffer size	1,000,000
Number of hidden units	256
Entropy target	−dim(*A*) = −3
Nonlinearity	ReLU
Target update interval	1
Target smoothing coefficient *τ*	5 ⋅ 10^–3^
Time step	10 s
Episode length	600 s = 10 min

**TABLE 3:** Training parameters for the PPO algorithm. For the RL hyperparameters and the environment parameters, see Table 2.

Parameter	Value
Horizon	60
Learning rate	5 ⋅ 10^–5^
Number of epochs	500
Clipping parameter	0.2
Number of hidden units	256
Batch size	128
Nonlinearity	ReLU

### 3.5 PID controller

To evaluate the performance of the DRL algorithms, a simple PID controller is implemented and interfaced with Basilisk. The PID controller is tuned with a spacecraft mass of m = 300 kg. This represents roughly the median of the considered mass range while being slightly optimized for lower masses. This optimization is considered because high forces have a stronger effect on low masses. To tune the PID controller, the parameters are first approximated by hand, after which a Nelder and Mead (1965) optimization is employed to find the values for which the return is maximized. The parameters then correspond to: *k*_*p*_, *k*_*i*_, *k*_*d*_ = (0.36, 0.00015, 14.02). As input to the PID controller, the attitude error $\sigma_{B/N}$ is used. The PID controller has a control frequency of 1 Hz. In correspondence to the DRL algorithms, the PID controller outputs a 3-dimensional torque on the reaction wheels. The PID controller is used for comparison with the DRL algorithms in the following section.

## 4 Results

In this section, the respective algorithms are evaluated and compared in terms of their achieved return and the final attitude error. First, the evaluation method is presented, and second, the results are discussed. The evaluation shows that the DRL algorithms with stacked observations outperform both the DRL without stacked observations and the classical PID controller.To evaluate the different algorithms, the trained agent is tested in the environment for 1,000 episodes. The initial and desired attitudes are the same as those used in the training. For each episode, the mass is uniformly sampled between 10 kg and 1,000 kg. The 1,000 episodes are then evaluated on the basis of the following metrics: **Return** ***R*** **:** The mean return reached in each episode, including the standard deviation. $|\sigma_{B/N\mathrm{fi}nal}|:$ The mean norm and standard deviation of the final 3D attitude error vector. $\sigma_{B/N\mathrm{fi}nal}^{i}:$ All final attitude vectors are transformed into one list by concatenating their components.Then, the mean and standard deviation of that list is calculated. **Confidence Interval (CI) for** $\sigma_{B/N\mathrm{fi}nal}^{i}:$ Gives an indication of where the final attitude error for all components will lie, with 95% confidence. **Settling time:** The mean and standard deviation of the time the satellite takes to get and keep the attitude error norm below 15°.

The results of the training metrics are listed in Table 4.

**TABLE 4:** Evaluation metrics for the SAC, PPO, and PID algorithms.

	Return	$|\sigma_{B/N\text{final}}|\;\left[{}^{\circ}\right]$	$\sigma_{B/N\text{final}}^{i}\;\left[{}^{\circ}\right]$	CI for $\sigma_{B/N\text{final}}^{i}\;\left[{}^{\circ}\right]$	Settling time [s]
SAC	0.95 ± 0.98	5.36 ± 5.19	−1.96 ± 3.84	[−5.69, 1.82]	232.77 ± 74.97
PPO	1.16 ± 0.90	9.83 ± 1.78	1.68 ± 5.52	[−7.86, 7.85]	217.52 ± 81.91
SAC stacked	1.95 ± 0.69	3.92 ± 2.58	−0.38 ± 2.68	[−6.46, 4.31]	195.77 ± 61.80
PPO stacked	2.23 ± 0.76	4.53 ± 1.21	0.98 ± 2.53	[−3.84, 5.12]	166.48 ± 12.46
PID	−0.64 ± 4.30	6.77 ± 16.75	−0.12 ± 10.43	[−4.62, 2.11]	229.42 ± 48.54

**FIGURE 2:**
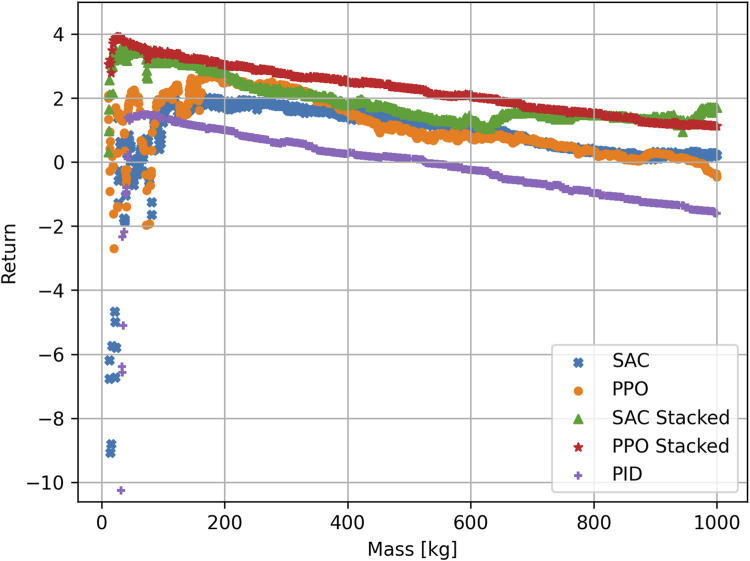
Return for the different algorithms plotted over the mass of the satellite. The PPO stacked algorithm achieves the best return overall. The algorithms with no stacked observations have difficulty in the low mass range. For visibility purposes, only returns to a minimum of −11 are shown.

The PPO algorithm with stacked observations achieves the best performance overall. Although the confidence interval of the attitude error $\left|\sigma_{B/N\text{final}}\right|$ is slightly lower for the classical PID controller, the standard deviation of the final attitude error is very high. This demonstrates that the RL stacked algorithms achieve a more robust control. Additionally, the algorithms with stacked observations have a faster settling time. Noticeably, both the SAC and the PPO algorithm have a larger CI for the final attitude error than the PID controller. This indicates that the PID algorithm provides results that are, in general, more consistent and, therefore, easier to predict than the other algorithms. Combined with the large standard deviation of the attitude error, this indicates that the PID controller achieves a stable and consistent result for most cases but cannot handle low masses. To further demonstrate this, the return in correlation to the mass is plotted for the different algorithms in Figure 2.

For most mass cases, the PID algorithm achieves a return within the same range. This explains the small CI of the final attitude error. The PID algorithm provides a very predictable return for masses m ≥ 100 kg, while for the DRL algorithms, the returns are higher in general but show a larger spread. The algorithms without stacked observations are not able to handle masses between 10 kg and 100 kg. Because the algorithms have no way to extract the mass from the observations, the agents try to generalize and optimize the actions for all masses. The algorithms maximize the expected return over all possible masses, which is why the return is slightly optimized for lower masses. By doing this, the agent must find a balance between applying a force that is too small to move the higher masses and applying a force that is too strong for the low masses. In the end, the actions are slightly more optimized toward lower masses because the negative effect of wrong actions is stronger here. This is evident in the shape of the return/mass curve, which starts negative and then reaches a maximum at approximately 200 kg, after which the return slightly decreases. The decrease in the return is due to the fact that the satellite needs a longer time to reach the desired orientation, with the force optimized for masses approximately 200 kg.

The SAC and PPO algorithms achieve slightly different curves. Although the mean return is in a similar range, the PPO algorithm performs slightly better at low masses. The optimization problem has different solutions: The SAC algorithm achieved a more generalized approach that achieves a consistent return for higher masses. The PPO algorithm achieves better results at low masses but performs worse at higher masses. The classical PID algorithm shows a very stable behavior for a wide array of masses but also fails to stabilize the satellite in the low mass range. This shows that a PID controller is not suitable for handling both low and very high masses because the tuning parameters would be vastly different.

**FIGURE 3:**
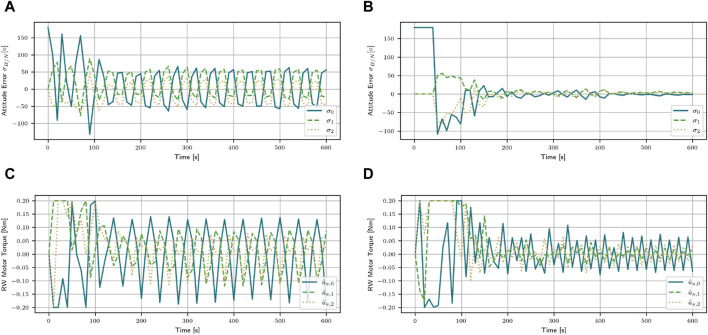
Attitude error and the applied motor torques for the mass of m = 15 kg with the SAC agent with and without stacked observations. Using stacked observations enables the agent to stabilize the satellite, even at low masses. **(A)** Attitude error for the SAC algorithm. **(B)** Attitude error for the SAC stacked algorithm. **(C)** Applied torques for the SAC algorithm. **(D)** Applied torques for the SAC stacked algorithm.

When using stacked observation, the agent is able to extract the dynamics of the spacecraft. By stacking the observations together, the agent can make a connection between the force applied and the reaction of the spacecraft over the course of five observations. This helps to stabilize the satellite, even at low masses. Instead of developing a generalized algorithm and applying the same actions to each mass, the agent learns to apply different actions depending on the mass. To further demonstrate the different behavior of the SAC agents with and without stacked observations, both agents are tested in one episode for a mass of m = 15 kg. The resulting evolution of the attitude error and the applied torques can be found in Figure 3. The agent without stacked observations reaches a return of *R* = −8.91, and the oscillating behavior around the desired orientation is clearly visible in Figure 3A. The agent applies torques that are optimized for masses approximately 200 kg. This results in the satellite overshooting the desired target. To mitigate this, the agent then applies a torque in the opposite direction. Again, this torque is too strong, causing another overshoot. The algorithm is unable to adapt to the fact that the mass is now significantly lower than those for which the algorithm optimized. The applied torque does not change in amplitude. On the other hand, the agent that uses stacked observations instead reaches a return of *R* = 2.95 and manages to stabilize the satellite with a stabilizing time of t = 260 s. After initially applying a large torque, the agent implements the feedback gained by the stacked observations to lower the force of the torque (Figure 3D). This allows the agent to stabilize the satellite at the desired orientation. The final attitude errors for SAC are [60.31, −27.02, −47.54]° and $|\sigma_{B/N\text{final}}|=81.41{}^{\circ}$ , SAC stacked instead reaches a final attitude error of [−0.77, 3.12, 1.14]° and $|\sigma_{B/N\text{final}}|=3.41{}^{\circ}$ . SAC stacked achieves a better result by 78°.

For further comparison, the PID controller is compared to the SAC stacked algorithm for m = 300 kg. This mass corresponds to the mass the PID controller was tuned with. This comparison will aid in explaining the difference between the two algorithms.

**FIGURE 4:**
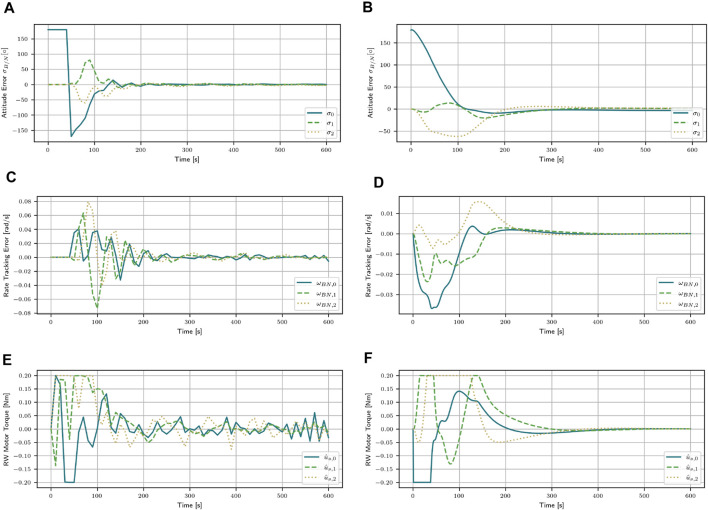
Attitude error, attitude rate error, and the applied motor torques for the mass of m = 300 kg with the SAC agent with stacked observations and the PID controller. **(A)** Attitude error for the SAC stacked algorithm. **(B)** Attitude error for the PID controller. **(C)** Attitude rate error for the SAC stacked algorithm. **(D)** Attitude rate error for the PID controller. **(E)** Applied torques for the SAC stacked algorithm. **(F)** Applied torques for the PID controller.

Both algorithms bring the attitude error close to zero. The SAC stacked algorithm reaches a return of *R* = 2.16, while the PID controller reaches *R* = 0.65. The final attitude error of the SAC stacked algorithm is [0.37, −1.94, −2.57]° and $|\sigma_{B/N\text{final}}|=3.24{}^{\circ}$ , while the PID controller achieves [−3.41, 1.23, 2.09]° and $|\sigma_{B/N\text{final}}|=4.19{}^{\circ}$ , respectively. The SAC stacked algorithm also brings the attitude error close to zero faster, achieving a settling time of 180 s, while the PID algorithm needs 206 s to achieve the desired attitude. The PID controller achieves smooth control by continuously adjusting its output based on the error between the desired and actual attitude. Once the final attitude is reached, the controller’s actions converge to zero, resulting in minimal torque applied. This behavior is a result of its feedback mechanism and predefined control parameters.In contrast, reinforcement learning agents use stochastic gradient descent to find the optimal policy. Therefore, it is only possible to approximate a certain output. As shown in Figure 4F, the SAC stacked agent cannot output the action [0, 0, 0]. For this reason, even if the attitude error is already very small, a small action is still carried out, which means that the satellite never comes to a complete rest. In contrast, the PID controller can output the action [0, 0, 0] and thus bring the attitude error very close to 0. This also explains the slight drop in the return for low masses in Figure 2 for the agents with stacked observations.

## 5 Conclusion and further study

This study demonstrated the great promise of DRL techniques, especially in combination with stacked observations. For scenarios where a high variation in mass is possible, such as active debris removal, the satellite ACS system must be highly adaptable for a wide range of possible masses. DRL algorithms without stacked observations are already able to handle the attitude control well, but they achieve similar results as a PID controller. Employing stacked observations significantly increases the performance of the DRL controller, especially for a low mass range. This technique enables the agent to extract mass information without measuring the mass directly. This is very valuable when dealing with the unknown space debris environment. Both the SAC and PPO algorithms achieved similar results.

These results establish a basis for further study. In the future, a higher varying mass range in combination with varying initial attitudes will be investigated. Combining these would lead to developing a robust attitude controller that can adapt its control based on the dynamics of the spacecraft. In this study, the mass variation was set before each episode. In a rendezvous maneuver, the mass would change from one time step to the next. The algorithm would have to adapt immediately. This change in mass during the episode will be investigated in future works.

## Data Availability

The raw data supporting the conclusion of this article will be made available by the authors, upon request.
